# Transcriptome Analysis of Hypoxic Lymphatic Endothelial Cells Indicates Their Potential to Contribute to Extracellular Matrix Rearrangement

**DOI:** 10.3390/cells10051008

**Published:** 2021-04-24

**Authors:** Jürgen Becker, Sonja Schwoch, Christina Zelent, Maren Sitte, Gabriela Salinas, Jörg Wilting

**Affiliations:** 1 Department of Anatomy and Cell Biology, University Medical School Göttingen, 37075 Göttingen, Germany; juergen.becker@med.uni-goettingen.de (J.B.); sschwoc@gwdg.de (S.S.); christina.zelent@med.uni-goettingen.de (C.Z.); 2 NGS-Integrative Genomics Core Unit (NIG), Institute of Human Genetics, University Medical Center Göttingen, 37075 Göttingen, Germany; Maren.Sitte@med.uni-goettingen.de (M.S.); Gabriela.Salinas-Riester@medizin.uni-goettingen.de (G.S.)

**Keywords:** hypoxia, extracellular matrix, hyaluronan, collagen, fibrillogenesis, elastin, fibulin 5, ceruloplasmin, TGFΒ, fibromodulin, ADAMTS15, GLUT3

## Abstract

Lymphedema (LE) affects millions of people worldwide. It is a chronic progressive disease with massive development of fibrosclerosis when untreated. There is no pharmacological treatment of lymphedema. The disease is associated with swelling of the interstitium of the affected organ, mostly arm or leg, impressive development of adipose tissue, fibrosis and sclerosis with accumulation of huge amounts of collagen, and *Papillomatosis cutis*. Malnutrition and reduced oxygenation of the affected tissues is a hallmark of lymphedema. Here, we investigated if the hypoxia of lymphatic endothelial cells (LECs) might contribute to fibrosis. We applied RNASeq and qPCR to study the concordant changes of the exome of three human foreskin-derived LEC isolates after 4 days of hypoxia (1% O_2_) vs. normoxia (21% O_2_). Of the approximately 16,000 genes expressed in LECs, 162 (1%) were up- or down-regulated by hypoxia. Of these, 21 genes have important functions in the production or modification of the extracellular matrix (ECM). In addition to the down-regulation of elastin, we found up-regulation of druggable enzymes and regulators such as the long non-coding RNA H19, inter-alpha-trypsin inhibitor heavy chain family member 5 (ITIH5), lysyl-oxidase (LOX), prolyl 4-hydroxylase subunit alpha 1 (P4HA1), procollagen-lysine 2-oxoglutarate 5-dioxygenase 2 (PLOD2), and others that are discussed in the paper. Initial lymphatics do not produce a continuous basement membrane; however, our study shows that hypoxic LECs have an unexpectedly high ability to alter the ECM.

## 1. Introduction

Increased production and insufficient clearance of interstitial fluid, and the various substances dissolved in it, are the major causes for edema formation. When edema is due to the insufficient clearance via the lymphatic vascular system, it is defined as lymphedema. In case of malignant lymphedema, tumor cells have occluded the lymphatics and prevent lymph flow. However, lymphedema can be caused by numerous internal and external noxae [1]. Thereby, primary lymphedema is due to mutations in lymphangiogenesis genes, which control the development and differentiation of initial lymphatics, collectors, trunks, and lymph nodes [2]. Secondary lymphedema may be caused by numerous factors, including cancer, cancer therapy, surgery, infection, trauma, obesity, and others [1,3].

Of note, lymphedema (LE) is a chronic disease, which, when untreated, progresses into stage-III LE. Due to massive tissue hyperplasia of the affected region (often arm or leg), stage-III LE has previously been called ‘elephantiasis’. This term emphasizes the conspicuous alterations provoked by LE but lacks the necessary respectful treatment of patients. Stage-III LE is characterized by a hard, distorted swelling of the affected area and typical verrucous skin alterations. Starting initially with an increase in free interstitial fluid, advancement into stage-II and stage-III LE is characterized by molecular alterations of the interstitium, accumulation of adipose tissue (‘lymph makes you fat’) [4,5], increase in connective tissue and extracellular matrix (fibrosis/sclerosis), development of lymphatic cysts, trophic changes of the epidermis (including verrucous protuberances), reduced immune responses, and painful changes in the musculoskeletal system [1]. While some patients develop great amounts of adipose tissue, others have a predisposition for fibrosclerosis formation. The reasons behind this differential behavior are unknown.

The intercellular space is the first compartment affected by LE. Initially, there is overload with free interstitial fluid, which can be drained easily by elevation of the affected limb or manual lymph drainage. However, fluid is then efficiently bound by increasing amounts of the glycosaminoglycan hyaluronan [6], forming an interstitial gel. Hyaluronan (HA) turnover in skin is usually very high, and the initial lymphatics are equipped with the HA receptor LYVE1 for the removal of HA via the lymph and its degradation in lymph nodes and liver [7,8]. However, catabolism of HA takes place both by drainage via the lymphatic system and local degradation [9]. During chronic progression of LE into stage-II and stage-III, there is a marked increase in collagen fiber production and deposition, resulting in the typical picture of fibrosclerosis and considerable thickening of the corium. Thereby, type-I and type-III collagen fibers have been identified, which are also deposited as a dense layer around the initial lymphatics in the corium [10]. The massive increase of interstitial spaces, the rock-hard fibrosclerosis, and the basal lamina-like fiber deposition around initial lymphatics results in malnutrition and critically reduced oxygenation of cells, including lymphatic endothelial cells (LECs). It is well known that hypoxia is a potent regulator of cellular functions [11]. Key regulators of oxygen homeostasis are the transcriptionally active hypoxia-inducible factor(s) (HIF) and their regulators the prolyl hydroxylase(s) (PHD). Enhanced lymphendothelial expression of Hif1α in clinical lymphedema and important functions for both Hif1α and Hif2α during lymphatic vascular development and regeneration in mice have been found [12,13]. 

Here, we asked the question if chronic hypoxia of human LECs in vitro regulates genes that may contribute to the sequelae of lymphedema, especially fibrosclerosis. Effects of hypoxia have previously been studied in LECs using microarray techniques for 22,000 genes [14]. However, these authors have used human microvascular endothelial cells (HMVECs), which usually are a mixture of blood vascular endothelial cells (BECs) and LECs. With RNASeq, we observed the hypoxia-induced regulation of 162 genes, including two pseudogenes and 11 long non-coding (lnc) RNAs, which are consistently regulated in three juvenile foreskin LEC isolates. Here, we focus on genes involved in extracellular matrix (ECM) production, stabilization, and degradation. We identified 21 genes, 18 up- and 3 down-regulated, which will be presented and discussed.

## 2. Materials and Methods

### 2.1. Cell Culture

**Figure 1:**
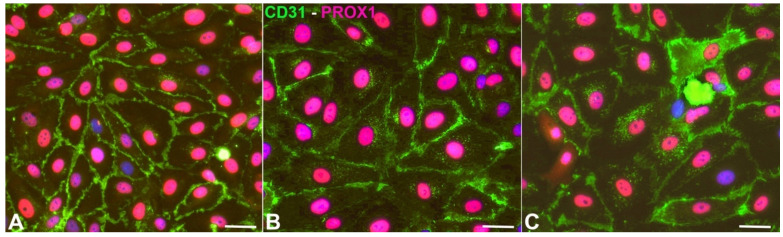
Expression of CD31 (green) and PROX1 (magenta) in HD-LECc5 (**A**), HD-LECc6 (**B**), and HD-LECc7 (**C**). Bar = 15 µm. Pictures were acquired with AxioImager Z.1 (Zeiss, Göttingen, Germany).

Normal juvenile foreskin-derived LECs were bought from PromoCell (Heidelberg, Germany). There, the cells are routinely analyzed by flow cytometry and immunofluorescent staining. More than 95% of the cells are CD31 positive and podoplanin positive. Cells were cultured in endothelial growth medium (MV2) with supplements and fetal bovine serum (PromoCell, Heidelberg, Germany) and 1% penicillin/streptomycin (Thermo-Fisher, Darmstadt, Germany). In order to select the purest cultures, we tested the cells for PROX1 expression in combination with CD31, which is the combination of markers that identifies LECs of all ages [15] (Figure 1). Seeking the highest purity, three out of six cell cultures were chosen for the hypoxia experiments. The cell isolates were designated HD-LECc5, HD-LECc6, and HD-LECc7 (or just LEC5, LEC6, and LEC7). The purity of the cultures was close to 100%. For the experiments (n = 3 for each isolate), confluent cells of passage 5 were cultured at 37 °C and 5% CO_2_ at normoxia (21% pO2; Sanyo CO2 incubator) and hypoxia (1% pO2; Baker Ruskinn InvivO2). The experiments lasted 4 days to mimic chronic hypoxic conditions. Cells were washed in PBS, lysed on the dish with peqGOLD TriFast (VWR, Darmstadt, Germany), and harvested using a cell scraper. Experiments with all three cell isolates were repeated three times. All experiments were validated by qPCR for typical hypoxia-regulated gene expression. RNA and protein were isolated in subsequent steps, according to the manufacturer’s protocol. 

### 2.2. Immunocytology

Cells were seeded on chamber slides (BD, Heidelberg, Germany). After 24 hours (h), immunostaining was performed as published previously [16]. Briefly, after 1 min fixation with 4% paraformaldehyde (PFA), cells were washed with PBS and incubated in PBS containing 5% bovine serum albumin (BSA). Then, 0.1% Tween/PBS (30 s) was applied, followed by immunostaining. Anti-human PROX1 antibody (1:200, ReliaTech, Wolfenbüttel, Germany), and anti-human CD31 antibody (1:50; BD, Franklin Lakes, NJ, USA) were incubated for 1h, cells were rinsed with PBS, and secondary antibody staining with Alexa594-conjugated goat-anti-rabbit and Alexa488-conjugated goat-anti-mouse antibodies (1:200; Thermo Fisher Scientific, Waltham, MA, USA) was performed. Antibodies were diluted in PBS containing 5% BSA. Cells were counterstained with DAPI and analyzed with AxioImager Z.1 (Zeiss, Göttingen, Germany) and processed with AdobePhotoshop (Adobe, San Jose, CA, USA).

### 2.3. Real-Time RT-PCR (qPCR)

Methods for qPCR were basically the same as described [17]. Primers are listed in Table 1. In brief, total RNA was isolated directly from the culture plates using PeqGold TriFast (VWR, Darmstadt, Germany) and 2 µg were transcribed using the Qiagen Omniscript reverse transcriptase (QIAGEN, Hilden, Germany). QPCR was performed on a MIC device (BMS, Upper Coomera, QLD, Australia) using Fast SYBR Green Mastermix (Thermo Fisher Scientific, Darmstadt, Germany). All kits were used as suggested by the manufacturers. All samples were tested in triplicates, and relative expression was determined using the mean Ct of the triplicates and normalization to β-actin according to the 2^−ΔΔCT^-method [18]. Plots were created using Microsoft Excel (Microsoft, Redmont, WA, USA).

### 2.4. Transcriptome and Bioinformatic Analysis

*RNA-Seq library preparation*: Quality and integrity of RNA was assessed with the Fragment Analyzer (Advanced Analytical Technologies, Heidelberg, Germany) by using the standard DNF-471 Sensitivity RNA Analysis Kit (Cultek, Madrid, Spain). All samples selected for sequencing exhibited an RNA integrity number over 8. RNA-seq libraries were performed using 500 ng total RNA of a non-stranded RNA Seq, massively-parallel mRNA sequencing approach from Illumina (TruSeq stranded total RNA Library Preparation, Illumina, San Diego, CA, USA). Libraries were prepared on the Biomek FXP automation workstation (Beckman Coulter, Brea, CA, USA) as described previously [19]. For accurate quantitation of cDNA libraries, a fluorometric-based system, the QuantiFluor dsDNA System (Promega, Heidelberg, Germany), was used. The size of final cDNA libraries was determined by using the dsDNA 905 Reagent Kit and the Fragment Analyzer from Advanced Bioanalytical (Heidelberg, Germany) exhibiting a sizing of 300 bp in average. Libraries were pooled and sequenced on the Illumina HiSeq 4000 (Illumina, San Diego, CA, USA) (SE; 1 × 50 bp; 30–35 Mio reads/sample).

Sequence images were transformed with Illumina software BaseCaller (Illumina, San Diego, CA, USA) to BCL files, which were demultiplexed to fastq files with Bcl2Fastq v2.17.1.14 (Illumina, San Diego, CA, USA). The quality check was done using FastQC (Andrews, Simon. “FastQC a quality-control tool for high-throughput sequence data 2014; version 0.11.5, Babraham Bioinformatics, www.bioinformatics.babraham.ac.uk, accessed on 20 March 2020).

*Mapping and Normalization*: Sequences were aligned to the genome reference GRCm38 (mm 10) sequence using the STAR aligner (Author: Alex Dobin, dobin@cshl.eduhttps://groups.google.com/d/forum/rna-star). As described before [20], read counting was performed using featureCounts (https://bioinformaticshome.com/tools/rna-seq/descriptions/FeatureCounts.html). Read counts were analyzed in the R/Bioconductor environment (version 3.4.2, www.bioconductor.org) using the DESeq2 package version 1.14.1. Candidate genes were filtered using an absolute log2 fold-change >1 and FDR-corrected *p*-value < 0.05. Gene annotation was performed using *Homo sapiens* entries via biomaRt R package version 2.32.1 (www.bioconductor.org).

## 3. Results

**Figure 2:**
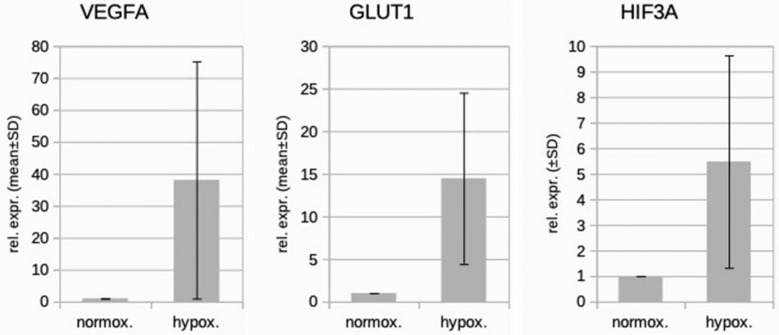
Real-time PCR analysis of HD-LECc5, HD-LECc6, and HD-LECc7 under normoxic (21% O_2_) and hypoxic (1% O_2_) conditions. Mean values from the three independent LEC donor samples are shown as bars; standard deviation as error bar. Mean values for normoxia were set to 1 to make samples comparable with the 2^−ΔΔCT^-method. Note the up-regulation of *VEGF-A*, *GLUT1*, and *HIF3A* by hypoxia. Calculations and plot were performed using Microsoft Excel 16 (Microsoft, Redmond, WA, USA).

**Figure 3:**
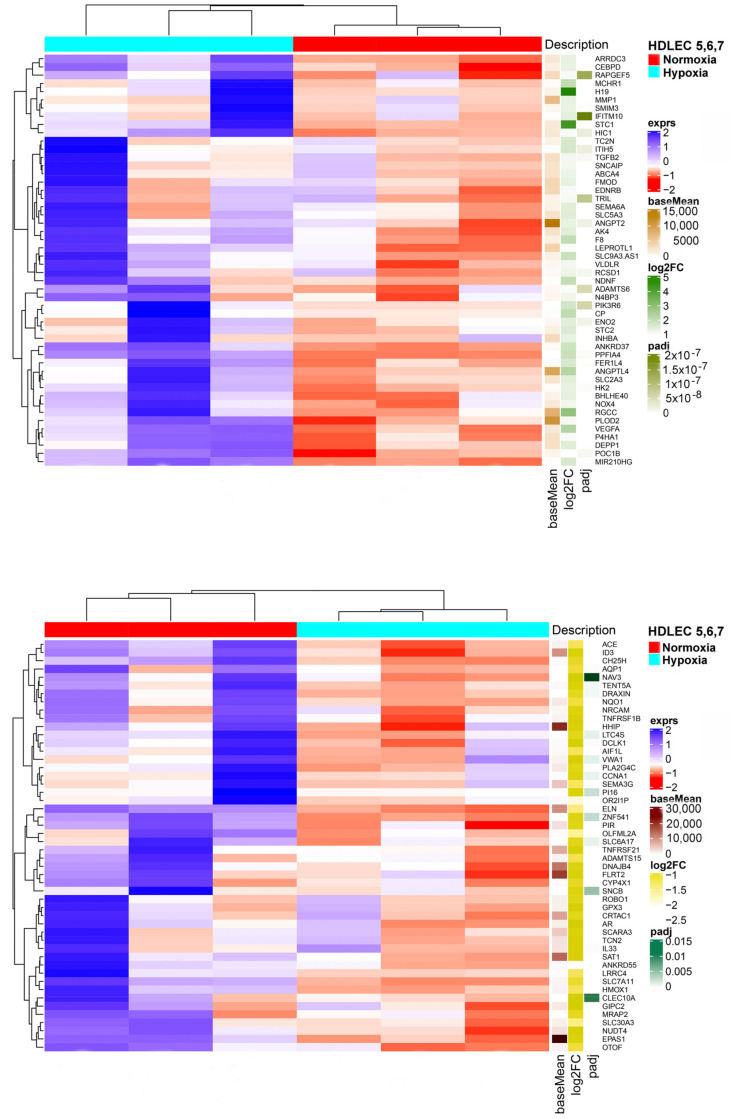
(**A**) Heatmap of the 50 most up-regulated differentially expressed genes in HD-LECs under hypoxia compared to normoxia. Produced with R version 3.6.3 and package ComplexHeatmap v2.0.0. (www.bioconductor.org/packages/release/bioc/html/ComplexHeatmap.html). (**B**) Heatmap of the 50 most down-regulated differentially expressed genes in HD-LECs under hypoxia compared to normoxia. Produced with R version 3.6.3 and package ComplexHeatmap v2.0.0. (www.bioconductor.org/packages/release/bioc/html/ComplexHeatmap.html).

**Figure 4:**
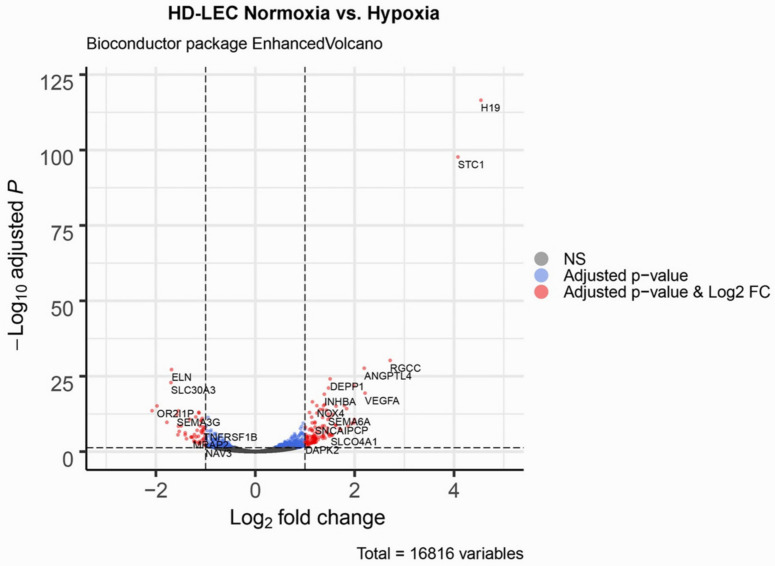
Volcano plot of significant genes. The dashed horizontal line signals statistical significance threshold (adjusted *p*-values ≤ 0.05). Two vertical lines show the threshold of log2 fold-change ≥1 and ≤−1. The colors indicate for the genes whether they have adjusted *p*-values ≤ 0.05 (blue), or they are significantly regulated and have a log2 fold-change ≥1 or ≤−1 (red). Produced with EnhancedVolcano v1.2.0. (www.bioconductor.org/packages/release/bioc/html/EnhancedVolcano.html).

We studied three lymphatic endothelial cell isolates, each of them representing an individual donor. The isolates were designated HD-LECc5, HD-LECc6, and HD-LECc7 (or short: LEC5, LEC6, and LEC7) (Figure 1). Experiments with each isolate were repeated three times, and we always observed the characteristic up-regulation of *VEGF-A*, *GLUT1*, and *HIF3A* (Figure 2). We chose one experiment for RNASeq analysis. Of the 16,816 genes expressed in LECs, approximately 1% were hypoxia-regulated. Thereby, we only listed genes that were consistently and significantly regulated in all three isolates. We observed the regulation of 162 genes (112 up- and 50 down-regulated) including two pseudogenes and 11 long non-coding RNAs (lncRNA). A heatmap is presented for the 50 most highly up-regulated (Figure 3A) and down-regulated genes (Figure 3B). The distribution of significantly regulated genes is shown as a volcano plot (Figure 4). Data show the regulation of characteristic endothelial genes such as VEGF-A, angiopoietin-like 4 (ANGPTL4), and semaphorins (SEMA6A, SEMA3G). The most significantly up- or down-regulated genes are the lncRNA H19 and elastin (ELN), respectively, which are genes that are involved in ECM formation (Figure 4). Among the regulated genes, we also found classical HIF-1 targets such as EGLN3 (Egl-9 Family Hypoxia Inducible Factor 3 = PHD3), which mediates a negative feedback after HIF-induction [21]. Additionally, we observed up-regulation of Solute Carrier Family 2 Member 3 (SLC2A3 = GLUT3), a hypoxia-regulated glucose carrier predominantly found in neurons, which also is a bidirectional transporter of dehydroascorbic acid (vitamin C). Among its multiple functions, vitamin C is an important coenzyme for prolyl-4-hydroxylases and well known for its function in collagen synthesis [22]. 

**Figure 5:**
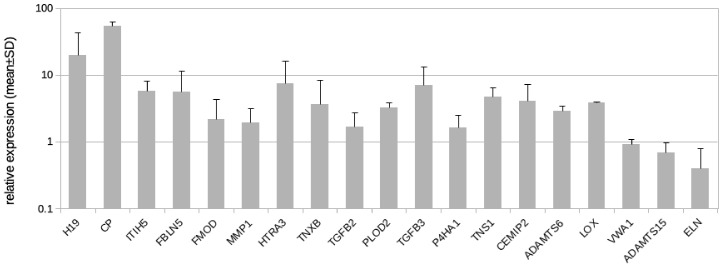
Real-time PCR analysis of HD-LECc5, HD-LECc6, and HD-LECc7 under hypoxia (1% O_2_); logarithmic scale. Mean value for the normoxic condition for each gene was set to 1 to make samples comparable with the 2^−ΔΔCT^-method. Mean values from the three LEC donor samples are shown as bars; standard deviation as error bar. Primer design for CHST2 did not reveal specific probes. For H19, the design of exon-spanning primers was not possible. Calculations and plot were performed using Microsoft Excel 16 (Microsoft, Redmond, WA, USA).

Here, we followed the question if LECs might contribute to the development of fibrosclerosis, which is characteristic for the advancement of lymphedema into stage-II and stage-III. In addition to GLUT3, we identified 19 protein coding genes and one lncRNA regulated by hypoxia in LECs with the potential to alter the ECM (Table 2). Our qPCR studies revealed results very similar to the RNASeq data (Table 2, Figure 5), with the exception of von Willebrand factor A domain containing 1 (VWA1), which was not regulated according to the qPCR data (Figure 5). In addition to the regulated genes, we studied the complete list of LEC-expressed genes with a focus on ECM genes and especially basal lamina components. Data show that LECs in vitro highly express basal lamina components such as type-IV collagen, laminin, fibrillin, and perlecan (Table 3). Below, all regulated molecules presented in Table 2 are discussed.

## 4. Discussion

The protein coding gene with direct ECM stabilizing functions most highly up-regulated by hypoxia in this list is inter-alpha-trypsin inhibitor heavy chain family member 5 (ITIH5). It contributes to ECM and hyaluronan (HA) stabilization and may therefore act as a tumor suppressor [23]. Of note, ITIHs (5 family members) are covalently linked to HA, which is abundantly produced in stage-I LE. ITIH5 is the major family member expressed in human skin, and it was suggested to be predominantly produced by dermal fibroblasts. It is up-regulated in inflammatory skin diseases. Interestingly, Itih5(−/−) mice reveal a significantly altered epidermal structure [24], which is somewhat reminiscent of the *papillomatosis cutis* found in stage-III LE. Increased expression of ITIH-5 in adipose tissue in obesity seems to be a direct link between obesity and LE aggravation [25]. Additionally, a function for ITIH-5 has been described for the induction of trans-differentiation of fibroblasts into myofibroblasts [26], which are the dominant cell type in scars. A selective ITIH5 inhibitor could be highly relevant for the resolution of fibrosis.

Fibulin 5 (FBLN5) was highly up-regulated in hypoxic LECs (Table 2). Fibulin is a secreted ECM protein with a typical integrin-binding Arg-Gly-Asp (RGD) domain and calcium-binding EGF-like domains. It thereby serves as an adhesion molecule, and it is highly expressed in the basal lamina of arterial endothelial cells [27]. It is associated with aortic dissection and *cutis laxa* [28,29,30]. FBLN5 is an elastin-binding protein [31] and was also shown to be up-regulated by hypoxia in bovine aortic endothelial cells (BAECs) and human umbilical vein endothelial cells (HUVEC) [32]. It is likely that fibulin 5 is a major constituent of the basal lamina that develops around initial lymphatics during LE progression. 

We observed significant up-regulation of fibromodulin (FMOD) by hypoxia. FMOD is a keratan sulfate proteoglycan, which has a primary role in collagen fibrillogenesis (Table 2). Its interactions with collagen affect collagen cross-linking, packing, and fibril diameter. Thereby, the cross-linking activity is accompanied by the activity of lysyl-oxidase (LOX) [33], which we found up-regulated in hypoxic LECs as well. Of note, in LE, the diameter of collagenous fibers increases to 40–400 nm in contrast to 25–200 nm in normal skin, and more collagen can be found showing cross-striations of 80–120 nm periodicity instead of 64 nm of normal type-1 collagen [10,34]. Similar to our studies, a potential function for FMOD in scar formation was found in liver and pancreas [35,36], but in contrast, reduced expression of FMOD was described in hypertrophic dermal scars [37]. Crosslinking of fibrillar collagens and elastin is carried out by the LOX and LOX-like family of extracellular enzymes. LOX is a copper-dependent amine oxidase that produces highly reactive aldehydes from lysine for spontaneous crosslinking. LOX activation in vivo appears to require ceruloplasmin (CP) [38], which is also up-regulated in hypoxic LECs (Table 2). CP is a copper-binding glycoprotein responsible for almost 95% of serum copper transport [39].

Another enzyme critical for collagen stability, and up-regulated in hypoxic LECs, is prolyl 4-hydroxylase subunit alpha 1 (P4HA1). It controls the triple-helical structure of procollagen and is a key enzyme in collagen synthesis [40,41].

In fetal mice, FMOD was shown to reduce scare formation and even to be required for scar-free wound repair ad integrum, which correlated with decreased expression of transforming growth factor-β1 (Tgf-β) in various rodent models of tissue repair [42]. However, it needs to be further investigated if there might exist species-specific or model-specific differences. In human liver and pancreas fibrosis FMOD is highly expressed [35,36]. We found the up-regulation of FMOD in hypoxic human LECs, and this coincided with higher LOX, TGF-β2, and TGF-β3 expression. The up-regulation of these collagen-stabilizing factors clearly shows that LECs are an important regulator of the ECM. In concert, TGF-β has repeatedly been identified as a potent profibrotic factor and even as a master-regulator of fibrosis [43]. However, this seems to apply predominantly to TGF-β1 [44], but up-regulation of the ECM glycoprotein fibronectin by TGF-β2 has also been observed [45]. An important function for matrix stabilization becomes obvious by the fact that mutations in *TGF-β2* and *TGF-β3* have been identified causative for Loeys-Dietz-syndrome 4 and 5, which is characterized by thoracic aortic aneurysm, joint laxity, and scoliosis [46,47,48].

In a liver fibrosis model, a link between TGFβ and the lncRNA H19 has recently been found [49]. Of note, H19 is the RNA most highly up-regulated in our complete list. A positive correlation between H19 and fibrosis has been found in several organs including liver, lung, and kidney [49,50,51]. In liver, H19 regulates the trans-differentiation of hepatic stellate cells into myofibroblasts [50], which is an important step in the pathogenesis of cirrhosis, and the inhibition of H19 has been recommended for anti-fibrosis therapy. 

In addition to molecules that enhance ECM production in hypoxic LECs, we also observed the up-regulation of enzymes involved in ECM degradation. Thereby, HtrA serine peptidase 3 (HTRA3) can cleave several ECM proteoglycans, including decorin and the closely related biglycan, which bind type-I collagen fibrils and control their assembly [52,53]. The degradation of decorin and biglycan may be another reason for the abnormal structure of collagens seen in LE. The up-regulated matrix metalloproteinase 1 (MMP1; collagenase) is a zinc-dependent secreted proteinase involved in several physiological and pathological processes [54]. It cleaves the helical domain of the major types of collagen.

Of note, the function of MMPs goes beyond matrix degradation, and some MMPs even possess pro-fibrotic effects e.g., by activation of processes related to immunity, tissue repair, and remodeling [55]. A specific problem related to studies on MMP1 resides in the fact that rodents possess *MMP1* in duplicated form [56], and statements on the function of human MMP1 remain speculative. However, MMP1 was the most highly up-regulated gene found in activated human stellate cells, which represent myofibroblasts of cirrhotic liver [57].

A protease family related to MMPs is the ADAM metallopeptidase with thrombospondin type 1 motif (ADAMTS) family. Thereby, we found the up-regulation of ADAMTS6 and down-regulation of ADAMTS15 in hypoxic LECs. The ADAMTS family consists of at least 19 members of secreted and matrix-bound enzymes, and it is subgrouped according to their substrates into the aggrecanases/proteoglycanases (ADAMTS1, 4, 5, 8, 9, 15 and 20), the procollagen N-propeptidases (ADAMTS2, 3 and 14), the cartilage oligomeric matrix protein-cleaving enzymes (ADAMTS7 and 12), the von Willebrand factor proteinases (ADAMTS13), and orphan enzymes (ADAMTS6, 10, 16, 17, 18 and 19) [58]. The functions of ADAMTS6 are not well characterized, but they may reside in the field of immunology, as it is regulated by tumor necrosis factor-α [59]. The down-regulation of ADAMTS15 in hypoxic LECs may indicate an decreased degradation of aggrecan (the main proteoglycan of cartilage) and proteoglycans per se.

We found up-regulation of the carbohydrate sulfotransferase 2 (CHST2) in hypoxic LECs. There are only a few studies on CHST2. However, it was originally detected in vascular endothelial cells [60]. CHST2 encodes an enzyme involved in glycosaminoglycan (GAG) sulfation, which increases the water-binding capacity of GAGs. 

Tenascin-XB (TNXB) was up-regulated in hypoxic LECs. TNXB is a glycoprotein of the ECM, and it is preferentially located in the fibro-reticular lamina of the basement membrane [61]. It accelerates collagen fibril formation and assembly, and it is mutated in the classical-like forms of Ehlers–Danlos syndrome, which is characterized by hypermobility of joints and skin [62].

The up-regulated procollagen-lysine 2-oxoglutarate 5-dioxygenase 2 (PLOD2; lysyl hydroxylase 2) is a membrane-bound homodimeric enzyme, which catalyzes the hydroxylation of lysyl residues in collagen-like proteins [63]. This post-translational modification of lysin is important for the cross-linking and stabilization of collagen filaments. Mutations in the PLOD2 gene cause the Bruck syndrome, which is characterized by the unusual combination of skeletal changes resembling osteogenesis imperfecta with congenital contractures of the large joints [64].

The up-regulation of the protein tensin-1 (TNS1) may indicate an intensified interaction between LECs and the ECM in hypoxia. TNS1 localizes to focal adhesions and links the ECM (mainly fibronectin) with the actin filament system in the cytoplasm, which controls cell shape. TNS1 also contains an Src homology 2 (SH2)-domain, which is an important component of intracellular signal transduction pathways that control transcription [65,66]. TNS1 has been associated with increased ECM production in kidney disease [67] and myofibroblast differentiation in lung fibrosis, where TNS1 is up-regulated by TGF-β [68]. Of note, TNS1 was identified as one of the ten most significantly regulated genes in keloids, which are benign fibroproliferative tumors in abnormal wound healing [69].

The turnover of hyaluronan (HA) is very high and is estimated to be one-third per day [70]. The lymphatics possess an outstanding importance in the degradation and transport of HA, and the up-regulation of CEMIP2 (cell migration inducing hyaluronidase 2; also known as transmembrane protein 2; TMEM2) suggests that under hypoxia, the LECs try to further increase this function. CEMIP2 is a transmembrane protein acting as cell surface hyaluronidase, which cleaves high molecular weight hyaluronan into intermediate fragments. By interaction with CD44/LYVE1 on LECs, this facilitates transcellular transport into the lymph for the further degradation of HA in lymph nodes and liver [9,70]. In contrast to other hyaluronidases, CEMIP2 specifically cleaves HA, and it does not cleave dermatan or chondroitin sulfate [70]. An angiogenic function of CEMIP2 resides in the fact that vascular endothelial growth factors are stored in the ECM by binding to HA [71].

With RNASeq, we observed the down-regulation of VWA1 (von Willebrand factor A domain containing 1); however, with qPCR, we could not verify this finding. VWA1 is a disulfide-bonded, multimeric secreted glycoprotein. The number of studies on VWA1 is sparse (reviewed by [72]). It is highly expressed in cartilage, muscle, and the basement membrane of endothelial cells and may facilitate linkages with type VI collagen. However, the global knock-out of *Vwa1* in mice does not seem to induce failure in cartilage, muscle, or vessels, but in the ECM of peripheral nerves, which results in defects in fine motor coordination and nociception [73].

The important functions of elastic fiber networks, consisting predominantly of elastin (ELN) and fibrillin, are very obvious, and they are underlined by the fact that mutations in the *ELN* gene are the cause of several syndromes: Williams–Beuren syndrome, supravalvular aortic stenosis, and cutis laxa-autosomal dominant 1 [74,75,76,77]. We found significant down-regulation of ELN in hypoxic LECs, which can be very well correlated with the increasing tissue stiffness found in stage-II and stage-III LE. Loss of ELN may not only result in decreased elasticity of tissue but may as well stimulate proliferation. In Eln knock-out mice, it was shown that the loss of Eln in cultured arteries is *per se* sufficient to induce subendothelial cell proliferation. A comparable effect may also contribute to the chronic advancement of fibrous tissue in LE. 

The loss of ELN, which is preferentially colocalized to initial lymphatics, in advanced stages of LE, has been demonstrated by orcein staining. Thereby, the disappearance of ELN was mostly attributed to the upregulation of elastases by neutrophils, macrophages, or mast cells [78]. Our data suggest that down-regulation by LECs may be another important mechanism. While ELN is down-regulated in hypoxic LECs, the enzyme responsible for crosslinking of lysine residues in ELN, lysyl-oxidase (LOX), is upregulated and may then induce more efficient cross-linking of collagen fibrils. Down-regulation of ELN is also in contrast to the increase in fibulin-5 (FBLN5), which controls the aggregation and positioning of elastic fibers. In sum, this may result in uncontrolled matrix deposition and patterning.

## 5. Conclusions

Our data show that LECs in vitro express numerous ECM proteins including important basal lamina components. In vitro, hypoxia induces transcriptional changes in LECs that appear to be highly relevant for the advancement of fibrosis. The morphology of advanced lymphedema in skin is characterized by increased thickness of the dermis, increased numbers of matrix fibers, laceration of fibers, deposition of amorphous ECM, increased ECM deposition around nerves, blood vessels and lymphatics, development of a basement membrane around initial lymphatics, altered ultrastructure of collagen fibrils, and the dissociation and breakdown of elastic fibers [10]. Our data suggest that LECs possess a high potential to alter the composition of the ECM. Under hypoxia, they downregulate elastin and the proteoglycan degrading enzyme ADAMTS15. By stabilization of HA (by ITIH5), constant production of collagens, enhanced TGF-β signaling and collagen assembly (FMOD), up-regulation of stability promoting, and cross-linking enzymes such as PLOD2, P4HA1, and LOX, as well as increased cofactor availability (by CP and GLUT3), a stiffer collagen network may develop. The most significantly up-regulated gene encodes the lncRNA H19, which appears to possess a comprehensive function in fibrosis. Hopefully, the identified molecules may serve as targets for a pharmacological treatment to support comprehensive decongestive therapy (CDT) of lymphedema. 

## Figures and Tables

**Table 1:** Primers used for qPCR.

Name	Fwd 5′–3′	Rev 5′–3′
ADAMTS15	ACCTGAGTGGTTCATTGGGG	TCTCCTCCTCAGAAGGTCCG
ADAMTS6	ACCTGAGTGGTTCATTGGGG	TCTCCTCCTCAGAAGGTCCG
CEMIP2	TGGTCGCAAGCACAGACTAT	TGCAGGAACTGAGGGGTTTC
CP	GAGAGGCCCTGAAGAAGAGC	TGATGGTGTCTCCCACCTCT
ELN	CTTTCCCGGCTTTGGTGTCG	CCTGAGCTTCGGGGGAAATG
FBLN5	TCCTGCACCGACGGATATTG	GCTGGCAGTAACCATAGCGA
FMOD	AACCAGCTGCAGAAGATCCC	GCAGAAGCTGCTGATGGAGA
H19	GCACCTTGGACATCTGGAGT	GCCTACTCCACACTCCTCAC
HTRA3	CATACGGATGCGGACGATCA	GAATTCGGCGCAACCTCTTG
ITIH5	GTGGGGTCGCAGGAAGAG	AACAGTCTGACTTGCCTCGG
LOX	GGGTCTGAATCCCACCCTTG	AAAAACGGGGCTCAAATCACG
MMP1	CCAGGTATTGGAGGGGATGC	GTCCAAGAGAATGGCCGAGT
P4HA1	AGGATGAATGGGACAAGCCTC	GGGTCATGTACTGTAGCTCGG
PLOD2	GAGAAGCCCTCGAGCATCC	GGCTGACTGCATAAATCGATGG
TGFB2	ACAACACCCTCTGGCTCAGT	CTGTAGAAAGTGGGCGGGAT
TGFB3	ACCCAGGAAAACACCGAGTC	TTAGGGCAGACAGCCAGTTC
TNS1	CCATGTCTCTGGTGGGCAAA	TGGGAGGATTTGAGCTGTCC
TNXB	TCTGTCAGGCAGGAAACGAC	AGGTAGCTCCTTCTCCAGGG
VWA1	TTGTGGACGTGGATGACCTG	CTGGACGTGATCTCCGTGG

**Table 2:** List of 20 fibrosis-related genes significantly up-regulated or down-regulated (minus log2 FC) by hypoxia in LECs. The table contains gene annotations (name, chromosome, strand, and gene type), average expression in all samples (baseMean), control samples (baseMean Ctrl), and treatment samples (baseMean Treat), as well as statistics for the genes (log2-fold-change of RNASeq studies, p-values, and adjusted *p*-values). Also, qPCR validation is shown. For CHST2, primer design for qPCR did not reveal specific probes. For H19, design of exon spanning primers was not possible. FC = fold change; lncRNA = long non-coding RNA; SD = standard deviation.

Gene Name	Chromos.	Strand	Gene type	Base Mean	Base Mean Ctrl	Base Mean Treat	log2 FC	qPCR FC ± SD	*p* Value	Padj
H19	11	-	lncRNA	1102.56	49.10	2156.03	4.54	19.9 ± 23	2.21 × 10^−121^	3.00 × 10^−117^
CP	3	-	prot_coding	131.14	5.32	256.96	1.98	54.6 ± 7.7	9.70 × 10^−13^	2.86 × 10^−10^
ITIH5	10	-	prot_coding	149.79	43.16	256.41	1.69	5.7 ± 2.5	1.10 × 10^−10^	2.22 × 10^−8^
FBLN5	14	-	prot_coding	472.33	140.55	804.10	1.47	5.5 ± 6.0	1.19 × 10^−8^	1.64 × 10^−6^
FMOD	1	-	prot_coding	3576.51	1724.37	5428.65	1.40	2.2 ± 2.1	2.23 × 10^−19^	2.33 × 10^−16^
MMP1	11	-	prot_coding	5844.68	2513.30	9176.05	1.35	1.9 ± 1.3	1.23 × 10^−14^	4.64 × 10^−12^
CHST2	3	+	prot_coding	1493.45	745.04	2241.85	1.24	-	1.30 × 10^−9^	2.13 × 10^−7^
HTRA3	4	+	prot_coding	183.02	87.13	278.90	1.22	7.5 ± 8.8	4.99 × 10^−7^	4.48 × 10^−5^
TNXB	6	-	prot_coding	208.02	45.08	370.95	1.21	3.7 ± 4.8	9.67 × 10^−6^	6.07 × 10^−4^
TGFB2	1	+	prot_coding	2836.67	1540.02	4133.33	1.20	1.7 ± 1.0	3.40 × 10^−11^	7.56 × 10^−9^
PLOD2	3	-	prot_coding	9973.82	6024.54	13923.10	1.15	3.2 ± 0.6	2.78 × 10^−20^	3.14 × 10^−17^
TGFB3	14	-	prot_coding	106.46	40.28	172.64	1.15	7.0 ± 6.2	2.13 × 10^−5^	1.17 × 10^−3^
P4HA1	10	-	prot_coding	2797.71	1640.17	3955.25	1.15	1.6 ± 0.9	9.35 × 10^−13^	2.82 × 10^−10^
TNS1	2	-	prot_coding	6950.84	4073.20	9828.48	1.14	4.7 ± 1.7	7.92 × 10^−7^	6.59 × 10^−5^
CEMIP2	9	-	prot_coding	5188.67	2510.43	7866.90	1.14	4.1 ± 3.1	8.12 × 10^−8^	8.73 × 10^−6^
ADAMTS6	5	-	prot_coding	624.19	374.99	873.40	1.09	2.9 ± 0.5	2.73 × 10^−10^	5.13 × 10^−8^
LOX	5	-	prot_coding	3001.85	1871.25	4132.45	1.04	3.9 ± 0.1	9.24 × 10^−8^	9.71 × 10^−6^
VWA1	1	+	prot_coding	687.79	877.41	498.16	−1.02	0.9 ± 0.2	1.12 × 10^−5^	6.79 × 10^−4^
ADAMTS15	11	+	prot_coding	584.60	895.85	273.35	−1.49	0.7 ± 0.3	1.22 × 10^−11^	3.00 × 10^−9^
ELN	7	+	prot_coding	6210.97	965.37	2770.58	−1.69	0.4 ± 0.4	2.26 × 10^−31^	6.14 × 10^−28^

**Table 3:** List of ECM genes highly expressed in LECs in vitro. Number of reads in all samples (Human Dermal-LEC5,6,7) is shown under normoxia (21% O_2_) and hypoxia (1% O_2_). Note expression of typical basal lamina components, which are not significantly up- or down-regulated by hypoxia.

Gene-Id	Gene-Symbol	Chromos.	Gene-Name	LEC5; 21% O_2_	LEC6; 21% O_2_	LEC7; 21% O_2_	LEC5; 1% O_2_	LEC6; 1% O_2_	LEC7; 1% O_2_
ENSG00000110799	VWF	12	von Willebrand factor	110,056	74,476	31,043	92,868	45,799	56,732
ENSG00000110800	COL4A1	13	Collagen IV alpha 1 chain	40,704	48,506	74,087	49,831	39,451	66,389
ENSG00000110801	COL4A2	13	Collagen IV alpha 2 chain	29,111	40,049	66,375	37,650	35,392	78,698
ENSG00000110802	COL8A1	3	Collagen VIII alpha 1 chain	19,050	34,930	9414	16,103	17,801	10,441
ENSG00000110803	COL18A1	21	Collagen XVIII alpha 1 chain	29,847	10,505	17,796	38,463	10,204	17,105
ENSG00000110804	COL5A2	2	Collagen V alpha 2 chain	3820	3535	13,285	5904	3889	12,653
ENSG00000110805	COL4A5	X	Collagen IV alpha 5 chain	2114	676	2081	2145	864	1977
ENSG00000110806	COL6A2	21	Collagen VI alpha 2 chain	104	359	304	229	535	383
ENSG00000110807	COL5A1	9	Collagen V alpha 1 chain	2250	306	3762	4098	458	6698
ENSG00000110808	COL11A2	6	Collagen XI alpha 2 chain	41	155	46	48	87	38
ENSG00000110809	FBN1	15	Fibrillin 1	10,648	5180	3912	9795	7227	3071
ENSG00000110810	LAMA4	6	Laminin alpha 4	22,245	16,175	11,342	27,918	16,742	15,632
ENSG00000110811	LAMA5	20	Laminin alpha 5	2174	632	4070	2380	465	4739
ENSG00000110812	LAMA3	18	Laminin alpha 3	484	167	349	582	125	366
ENSG00000110813	LAMB1	7	Laminin beta 1	28,134	14,482	23,098	30,873	13,910	38,887
ENSG00000110814	LAMB2	3	Laminin beta 2	10,535	9591	4763	13,436	10,002	7752
ENSG00000110815	HSPG2	1	Perlecan	50,132	24,772	30,919	37,175	14,955	39,144
ENSG00000110816	FN1	2	Fibronectin	104,522	45,391	173,759	156,880	90,964	671,160

## Data Availability

All data are included in the manuscript.

## Abbreviations

ADAMTS6/15	ADAM metallopeptidase with thrombospondin type 1 motif 6/15
ANGPTL4	Angiopoietin-like 4
BEC	Blood vascular endothelial cell
CDT	Comprehensive decongestive therapy
CEMIP2	Cell migration inducing hyaluronidase 2; also known as: transmembrane protein 2
CHST2	Carbohydrate sulfotransferase 2
CP	Ceruloplasmin
ECM	Extracellular matrix
EGLN3	Egl-9 family hypoxia inducible factor 3 (=PHD3)
ELN	Elastin
FBLN5	Fibulin 5
FMOD	Fibromodulin
H19	Long non-coding RNA H19
HA	Hyaluronic acid
HD-LEC	Human dermal lymphatic endothelial cells
HIF	Hypoxia-inducible factor
HMVEC	Human microvascular endothelial cell
HTRA3	HtrA serine peptidase 3
ITIH5	Inter-alpha-trypsin inhibitor heavy chain family member 5
LE	Lymphedema
LEC	Lymphatic endothelial cell
lncRNA	Long non-coding RNA
LOX	Lysyl-oxidase
MMP1	Matrix metalloproteinase 1
P4HA1	Prolyl 4-hydroxylase subunit alpha 1
PHD	Prolyl hydroxylase
PLOD2	Procollagen-lysine 2-oxoglutarate 5-dioxygenase 2
SLC2A3	Solute carrier family 2 member 3 (= GLUT3)
TGFB1/2/3	Transforming growth factor-β1/2/3
TNS1	Tensin-1
TNXB	Tenascin-XB
VEGF-A	Vascular endothelial growth factor-A
VWA1	von Willebrand factor A domain containing 1
